# Enhancing local recurrence detection in patients with high-grade soft tissue sarcoma: value of short-term Ultrasonography added to post-operative MRI surveillance

**DOI:** 10.1186/s40644-023-00645-9

**Published:** 2024-01-19

**Authors:** Ho Young Park, Hye Won Chung, Min A Yoon, Choong Guen Chee, Wanlim Kim, Jong-Seok Lee

**Affiliations:** 1 Department of Radiology and Research Institute of Radiology, Asan Medical Center, University of Ulsan College of Medicine, 88, Olympic-ro 43-gil, Songpa-gu, Seoul, 05505 Republic of Korea; 2 Department of Orthopedic Surgery, University of Ulsan College of Medicine, Asan Medical Center, Seoul, Republic of Korea

**Keywords:** Soft tissue sarcoma, Ultrasonography, Local recurrence

## Abstract

**Background:**

Limited data exist on the optimal postoperative surveillance protocol for high-grade soft tissue sarcoma, particularly regarding the optimal imaging modality and imaging interval for detecting local recurrence. This study aimed to assess the benefit of short-term postoperative ultrasonography (USG) for detecting local recurrence in patients with high-grade soft tissue sarcoma.

**Methods:**

Patients with newly diagnosed high-grade soft tissue sarcoma who underwent surgical resection between January 2010 and June 2020 were included. Short-term USG was added to the follow-up protocol as a surveillance tool alongside routine magnetic resonance imaging (MRI). The primary outcome was the additional detection rate of short-term USG compared with routine MRI surveillance for early local recurrence detection. Subgroup analysis was performed to evaluate factors influencing USG detection rate. The additional detection rate of short-term USG for detection of metastatic lymph nodes was also evaluated. The secondary outcome was the false referral rate of short-term USG.

**Results:**

In total, 198 patients (mean age ± standard deviation: 52.1 ± 15.8 years; 94 women) were included. Local recurrence occurred in 20 patients (10.1%; 20/198). Short-term USG detected local recurrence in advance of routine MRI visits in 7 out of 198 patients, resulting in an additional detection rate of 3.5% (95% CI: 1.7–7.1%). Subgroup analysis showed no significant difference in the short-term USG detection rate based on initial tumor characteristics, and receipt of radiotherapy or chemotherapy. Short-term USG additionally detected five of seven patients with metastatic lymph nodes [2.5% (95% CI, 1.1–5.8%, 5/198)]. The false referral rate of short-term USG was 3.5% (95% CI: 1.7–7.1%; 7/198).

**Conclusions:**

Short-term USG as part of postoperative surveillance for high-grade soft tissue sarcoma can enhance early detection of local tumor recurrence and metastatic lymphadenopathy. Early detection of local tumor recurrence could lead to a prompt surgical resection and aid in local disease control.

**Supplementary Information:**

The online version contains supplementary material available at 10.1186/s40644-023-00645-9.

## Introduction

Soft tissue sarcomas (STS) are rare malignant tumors that primarily affect the extremities or trunk. This group of tumors consists of diverse histologic grades and types [1]. High-grade STS, as defined by the French Federation of Cancer Centers histologic grading system, have a higher likelihood of local recurrence compared with low-grade STS and often recur within three years after surgical resection [2, 3]. However, limited data exist on the optimal postoperative surveillance protocol, particularly regarding routine follow-up imaging for detecting local recurrence [4, 5].

Current guidelines differ slightly in their recommendations for the preferred imaging modality and follow-up interval [3, 6, 7]. Magnetic resonance imaging (MRI) is commonly recommended for local recurrence surveillance, although ultrasonography (USG) is also suggested as a potential alternative by the National Comprehensive Cancer Network (NCCN) and the British Sarcoma Group [6, 7]. Moreover, these guidelines recommend variable follow-up intervals of 3–6 months. Notably, the guidelines do not explicitly address the necessity of routine imaging surveillance for detecting local recurrence, instead using vague terms such as “perform local imaging if necessary.” The supporting evidence for this assertion primarily comes from previous studies, where local recurrences were predominantly detected through self- or physical examination [4, 8, 9]. Some studies even reported similar local recurrence detection rates and overall survival outcomes between intense imaging follow-up and routine follow-up groups [8, 10]. However, studies emphasizing the importance of physical examination did not incorporate regular imaging with MRI or USG. Furthermore, direct comparisons of detection rates or survival analyses between the two follow-up approaches have limitations due to the heterogeneity of STS, which exhibit various clinical and biological behaviors. Importantly, these studies did not consider the “time dimension,” which refers to how early intense follow-up can detect local recurrence compared with routine protocols within the same patient. Early detection of local recurrence can potentially improve disease-specific survival [11], making it crucial to determine how many patients can benefit from an intense imaging follow-up protocol for early detection.

Our institution has implemented an intense imaging follow-up approach for STS. This involves routine MRI scans at intervals of 6–12 months, complemented by short-term USG performed between MRI visits. USG offers several advantages as an imaging modality, including its accessibility and higher spatial resolution compared with MRI. This makes it well-suited for detecting superficial lesions, which are common in STS [12]. Furthermore, USG has the potential to assist in identifying metastatic lymph nodes. However, the increased frequency of USG use may also lead to a higher number of false-positive cases, necessitating unnecessary advanced imaging, biopsies, or even surgeries. Despite these considerations, there is a lack of evidence regarding the use of short-term USG in STS surveillance. Therefore, our objective was to assess the additional benefits of incorporating short-term USG into routine MRI surveillance for the detection of local recurrence in patients with high-grade STS following surgical intervention.

## Materials and methods

Ethical approval for this retrospective study was obtained from the institutional review board of our institution, and the need for informed consent was waived.

### Patients

Consecutive patients who underwent surgical resection for STS between January 2010 and June 2020 were identified through a retrospective review of our electronic database. Inclusion criteria consisted of patients who (1) were diagnosed with high-grade (grade 2 or 3) STS located in the extremity or superficial trunk, (2) underwent initial curative surgical resection (e.g., wide excision or amputation), and (3) followed our prescribed surveillance protocol with regular Magnetic Resonance Imaging (MRI) and USG screenings for local recurrence. Exclusion criteria included the following: (1) low-grade STS, (2) the presence of metastatic or locally recurrent disease at initial presentation, (3) a positive resection margin, (4) follow-up loss before completing our surveillance protocol, and (5) incomplete imaging studies (including pre- or postoperative studies) or reference standards.

### Postoperative surveillance for local recurrence

**Fig. 1:**
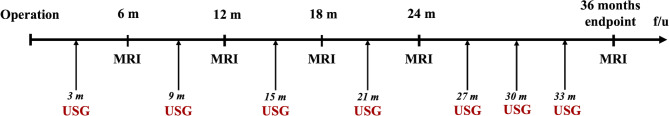
Imaging surveillance protocol used at our institution

Figure 1 illustrates our imaging surveillance protocol. The routine protocol involves MRI surveillance at 6-month intervals during the first two years, with subsequent annual MR follow-ups thereafter. Additionally, any palpable lesions identified through physical examination or self-palpation was further assessed using MRI. To evaluate the additional benefit of early detection of local recurrence, short-term USG was incorporated into the routine protocol between MRI visits. Our follow-up endpoint was set at three years, as most high-grade STSs locally recur within this timeframe [3].

### Imaging protocol

All USG examinations were conducted using a 12-MHz linear array or 9-MHz convex transducer on a USG system (Epiq 5G, Philips Healthcare). MRI scans were performed using either a 1.5-T scanner (Avanto, Siemens Healthinners) or a 3-T scanner (Ingenia or Achieva, Philips Healthcare, or Magnetom Skyra or Magnetom Vida, Siemens Healthineers). The choice of coil type, field of view, and matrix depended on the tumor location. The MRI imaging protocol included axial and either coronal or sagittal T1-weighted sequences, axial and either coronal or sagittal T2-weighted sequences, and axial fat-saturated T2-weighted sequences. Axial, coronal, and sagittal contrast-enhanced fat-saturated T1-weighted sequences were acquired in all patients after intravenous injection of contrast media (gadolilinum; Gadovist, Bayer, or Dotarem, Guerbet). The MR acquisition parameters were adjusted according to the anatomical region.

### USG and MRI analysis

Owing to the long recruitment period of 2010–2020, 49 musculoskeletal fellows in our radiology department conducted at least one USG examination of our patient cohort. All USG scans were double-checked by the staff members of our musculoskeletal radiology department (6 to 27 years of experience in musculoskeletal radiology).

In USG analysis, we considered a newly developed discrete low echogenic nodule larger than 0.5 cm as indicative of local recurrence. For MRI analysis, a newly developed discrete nodule larger than 0.5 cm showing high signal intensity on T2-weighted images, low signal intensity on T1-weighted images, and enhancement after contrast administration was considered as local recurrence. Additionally, newly observed fascial thickening and enhancement were deemed local recurrence, as these imaging patterns are commonly observed in undifferentiated pleomorphic sarcoma (UPS) or myxofibrosarcoma (MFS) [13, 14]. Two radiologists (H.Y.P. and H.W.C.), with 7 and 31 years of experience in diagnostic radiology, respectively, independently assessed all images. The readers were aware of the initial pathology but remained blinded to the occurrence of local recurrence. Furthermore, they were blinded to the results of each imaging modality when interpreting MRI or USG findings. In cases of ambiguous results, a consensus was reached between the two radiologists. Pathologic reports were used to confirm the presence of local recurrence. In case when pathologic confirmation was not performed, follow-up imaging in 3 ~ 6 month (either USG or MRI) was conducted to evaluate whether the size of the lesion increased or not. An unequivocal size increase was regarded as a local recurrence.

### Outcome

The primary outcome of this study was the additional detection rate of short-term USG in relation to routine MRI surveillance for the early detection of local recurrence in high-grade STS. Subgroup analysis was conducted to assess the influence of clinical and tumor characteristics on the detection rate of USG. The additional yield of short-term USG for metastatic lymph node detection was also evaluated. The secondary outcome was to evaluate the false referral rate of short-term USG.

### Statistical analysis

The detection rate, also referred to as the diagnostic yield, was calculated as the number of local recurrences divided by the total cohort, whereas the false referral rate was calculated as the number of false positives divided by the total cohort [15]. Fisher’s exact test was used to evaluate the detection rate of short-term USG in different subgroups based on tumor grade, size, depth, and whether patients received radiation or chemotherapy. *P*-values of < 0.05 were considered statistically significant. All statistical analyses were performed using R Statistical Software version 4.0.5.

## Results

### Patient demographics

**Fig. 2:**
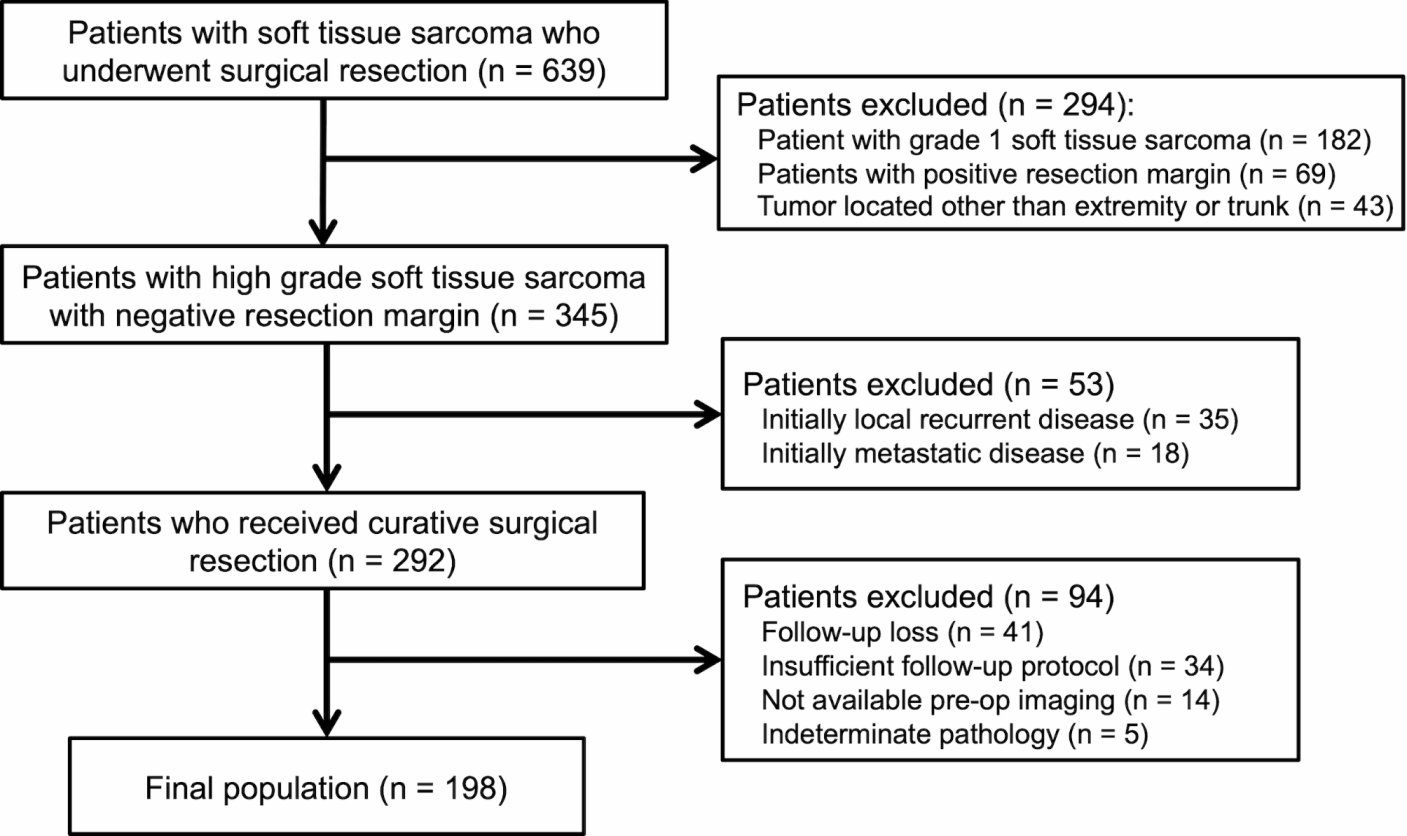
Flow diagram showing the patient inclusion process

A retrospective review of our database identified 639 patients who underwent surgical resection for STS at our institution. After excluding 347 patients based on specific criteria (182 patients with low-grade sarcoma, 69 with positive resection margin, 43 with tumor locations other than the extremities or superficial trunk, and 53 patients initially presenting with local recurrent or metastatic disease), as well as 94 patients for other reasons (75 patients lost during follow-up or receiving an insufficient follow-up protocol, 14 without pre-operation imaging, and 5 with indeterminate pathology), 198 patients were included for analysis (Fig. 2). The mean age [± standard deviation] of the included patients was 52.1 ± 15.8 years, with 104 men and 94 women. Patient characteristics are summarized in Tables 1 and 2. The majority of tumors were UPS, myxoid liposarcoma, and MFS (60.1%; 119/198). Lower extremities were the most common tumor locations (58.1%; 115/198), followed by the trunk and buttocks (24.2%; 48/198), and then the upper extremities (17.7%; 35/198). The tumors were distributed almost equally between intramuscular or intermuscular regions and dermis or subcutaneous layers. Grade 2 tumors were more prevalent than grade 3 tumors (61.6% vs. 38.4%).

**Table 1:** Patient demographics

Characteristics	Patients with high grade soft tissue sarcoma (n = 198)
Sex (n)	
Female	104 (59.6)
Male	94 (40.4)
Age (years)	52.1 ± 15.8 (mean ± standard deviation)
Radiation therapy	
No radiation therapy	65 (32.8)
Pre-op	1 (0.5)
Post-op	132 (66.7)
Chemotherapy^a^	
No chemotherapy	134 (67.7)
Pre-op	11 (5.6)
Post-op	63 (31.8)

Note: Data in parenthesis indicate percentages unless otherwise specified. ^a^Ten patients received both pre- and post-op chemotherapy.

**Table 2:** Tumor characteristics

Tumor	n = 198
Type of sarcoma	
Undifferentiated pleomorphic sarcoma	63 (31.8)
Myxoid liposarcoma	29 (14.6)
Myxofibrosarcoma	27 (13.6)
Synovial sarcoma	15 (7.6)
Fibrosarcoma	10 (5.1)
Malignant peripheral nerve sheath tumor	9 (4.5)
Dedifferentiated liposarcoma	8 (4.0)
Extraskeletal myxoid chondrosarcoma	5 (2.5)
Leiomyosarcoma	5 (2.5)
Others^a^	
Location	
Thigh	77 (38.9)
Calf and knee	34 (17.2)
Trunk^b^	32 (16.2)
Buttock	16 (8.1)
Upper arm	16 (8.1)
Forearm	14 (7.1)
Wrist and hand	5 (2.5)
Foot and ankle	4 (2.0)
Depth	
Intra or intermuscular	103 (52.0)
Dermis or subcutaneous	95 (48.0)
Size	
$<$5 cm	94 (47.5)
5 − 10 cm	62 (31.3)
10 − 15 cm	30 (15.2)
$\ge$15 cm	12 (6.1)
Grade	
Grade 2	122 (61.6)
Grade 3	76 (38.4)

Note: Data in parenthesis indicate percentages. ^a^Others include rhabdomyosarcoma (n = 4), clear cell sarcoma (n = 3), dermatofibrosarcoma protuberans (n = 3), Ewing sarcoma (n = 3), undifferentiated spindle cell sarcoma (n = 3), extraskeletal osteosarcoma (n = 2), angiosarcoma (n = 2), epithelioid sarcoma (n = 2), extraskeletal mesenchymal chondrosarcoma (n = 1), alveolar soft part sarcoma (n = 1), high grade pleomorphic sarcoma (n = 1), myofibroblastic sarcoma (n = 1), and spindle cell liposarcoma (n = 1). ^b^Trunk includes the anterior chest wall, back, and abdominopelvic wall.

### Local recurrence

In total, 20 out of 198 patients (10.1%) experienced local recurrence. The characteristics of these 20 patients are summarized in Table 3. The median interval between the operation and local recurrence was 18.5 months (range: 1–36 months), with the majority of recurrences occurring within 2 years (75.0%; 15/20). All instances of local recurrence were detected using either MRI or USG. Among these 20 cases, 6 patients (30%) initially presented with palpable lesions during physical examination.

**Table 3:** Patients with local tumor recurrence

Patient No. (Age, sex)	Diagnosis (Grade)	Location	Size (cm)/depth	Radiation/ chemotherapy	Time interval between operation and local recurrence (months)	Modality of detection	Local recurrence			
							Size (cm)	depth	USG findings	Palpable vs. nonpalpable at presentation
#1 (67, M)	UPS (Grade 3)	Trunk	4.0/intramuscular	+/+	9	Short-term USG	1.1	Subcutaneous	Well-defined homogeneous hypoechoic mass	Nonpalpable
#2 (61, F)	UPS (Grade 2)	Trunk	4.5/Subcutaneous	+/+	9	Short-term USG	2.2	Subcutaneous	Well-defined heterogeneous hypoechoic mass	Nonpalpable
#3 (38, F)	Epithelioid sarcoma (Grade 2)	Hand	1.3/Subcutaneous	+/-	12	Short-term USG	0.9	Subcutaneous	Partly defined heterogeneous hypoechoic mass	Nonpalpable
#4 (45, F)	Extraskeletal myxoid chondrosarcoma (Grade 2)	Buttock	10.5/Subcutaneous	-/-	12	Short-term USG	0.8	Subcutaneous	Two well-defined homogeneous hypoechoic mass	Nonpalpable
#5 (50, F)	Sclerosing epithelioid fibrosarcoma (Grade 3)	Calf	22.0/intramuscular	-/-	3	Short-term USG	1.7	Intermuscular	Well-defined homogeneous hypoechoic mass	Nonpalpable
#6 (54, M)	MFS (Grade 2)	Calf	4.3/Subcutaneous	+/-	30	Short-term USG	1.5	Subcutaneous	Partly defined homogeneous hypoechoic mass	Nonpalpable
#7 (57, F)	Synovial sarcoma (Grade 2)	Hand	2.8/intramuscular	+/+	30	Short-term USG	3.0	Intramuscular	Well-defined homogeneous hypoechoic mass	Nonpalpable
#8 (83, F)	UPS (Grade 2)	Thigh	3.4/Subcutaneous	+/-	27	Routine protocol (MR)	1.6	Subcutaneous	Well-defined heterogeneous hypoechoic mass	Nonpalpable
#9 (67, F)	UPS (Grade 3)	Axilla	3.7/Subcutaneous	+/-	27	Routine protocol (P/E)	0.8	Subcutaneous	Well-defined homogenous hypoechoic mass	Palpable
#10 (65, M)	MFS (Grade 2)	Calf	5.6/Subcutaneous	+/-	15	Routine protocol (P/E)	0.9	Subcutaneous	Well-defined heterogeneous hypoechoic mass	Palpable
#11 (61, F)	UPS (Grade 3)	Trunk	8.0/Intramuscular	-/-	15	Routine protocol (MR)	3.1	Subcutaneous	Well-defined homogenous hypoechoic mass	Nonpalpable
#12 (48, M)	UPS (Grade 3)	Thigh	11.2/Intramuscular	+/-	1	Routine protocol (P/E)	3.5	Intramuscular	NA^a^	Palpable
#13 (54, M)	UPS (Grade 3)	Calf	1.5/Subcutaneous	+/-	22	Routine protocol (P/E)	2.4	Subcutaneous	Well-defined homogenous hypoechoic mass	Palpable
#14 (58, F)	Angiosarcoma (Grade 2)	Forearm	3.5/Subcutaneous	+/+	21	Routine protocol (P/E)	NA^†^	Subcutaneous	NA^b^	Palpable
#15 (61, M)	Undifferentiated spindle cell sarcoma (Grade 3)	Thigh	11.5/Intramuscular	+/+	18	Routine protocol (MR)	5.0	Intramuscular	Well-defined homogenous hypoechoic mass	Non-palpable
#16 (47, F)	Undifferentiated spindle cell sarcoma (Grade 2)	Thigh	5.6/Subcutaneous	-/-	12	Routine protocol (MR)	1.3	Subcutaneous	Well-defined heterogeneous hypoechoic mass	Nonpalpable
#17 (55, M)	Undifferentiated spindle cell sarcoma (Grade 2)	Buttock	3.7/Subcutaneous	-/-	18	Routine protocol (MR)	1.7	Intramuscular	Well-defined homogenous hypoechoic mass	Nonpalpable
#18 (20, F)	Sclerosing/spindle cell rhabdomyosarcoma (Grade 3)	Thigh	11.9/Intramuscular	+/+	36	Routine protocol (MR)	1.2	Intramuscular	Well-defined homogenous hypoechoic mass	Nonpalpable
#19 (70, F)	MFS (Grade 2)	Buttock	10.7/Intramuscular	+/-	24	Routine protocol (MR)	6.0	Intramuscular	Partly-defined homogeneous hypoechoic mass	Nonpalpable
#20 (46, M)	Synovial sarcoma (Grade 3)	Thigh	8.2/Intramuscular	+/+	21	Routine protocol (P/E)	3.0	Intermuscular	Several well-defined homogeneous hypoechoic mass	Palpable

UPS: undifferentiated pleomorphic sarcoma; USG: ultrasonography; MFS: myxofibrosarcoma; P/E: physical examination; NA: not available.

^a^USG imaging findings were not available because only MRI was performed in this patient for the palpable lesion.

^b^The size of the local recurrence was not available in this patient because punch biopsy was performed for the palpable lesion before the imaging study.

### Additional yield of short-term USG for early detection of local recurrence and metastatic lymph nodes

**Fig. 3:**
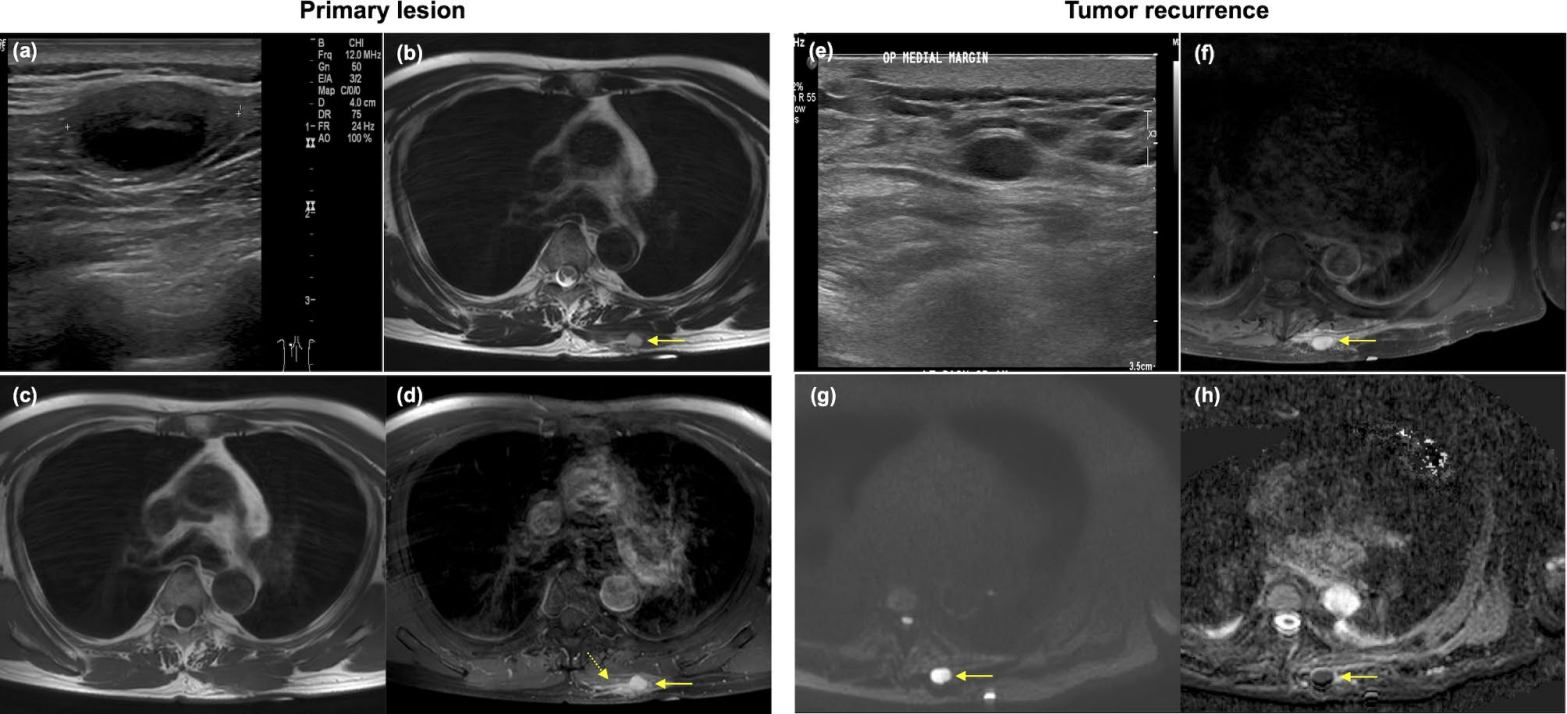
Early detection of local recurrence using short-term USG in a 67-year-old male with undifferentiated pleomorphic sarcoma. (**a**) Initial USG revealed a well-defined hypoechoic mass in the left trapezius muscle. (**b,c,d**) Axial T2-, T1-, and enhanced T1-weighted MR images show a well-defined T2 high signal intensity mass with enhancement in the left trapezius muscle, indicating a residual tumor (arrows). Ill-defined infiltration and enhancement are observed at the medial aspect of the tumor, representing postexcisional changes with or without tumor infiltration (dashed arrow). This lesion was a residual mass observed after excision at our dermatology department and pathologically confirmed as sarcoma. The patient subsequently underwent wide excision performed by an orthopedic oncologist. (**e**) On his 9-month postoperative USG, a newly detected 1.1-cm well-defined hypoechoic nodule was observed at the medial margin of the flap. Consequently, his MRI examination was rescheduled. (**f**) An enhanced T1-weighted MR image revealed a prominent enhancing mass with signal characteristics similar to those of the initial lesion. (**g,h**) High b-value (b = 1000 s/mm^2^) and apparent diffusion coefficient map images revealed diffusion restriction in the corresponding lesion (arrows). Wide resection was performed, and the lesion was pathologically confirmed as a recurrent tumor

Table 4 presents the detection rates of the routine protocol and short-term USG for local recurrence and metastatic lymph nodes. Short-term USG detected local recurrence in 7 out of 20 patients prior to their scheduled MRI visits, resulting in an additional yield for early detection of 3.5% (95% CI: 1.7–7.1%; 7/198). Following USG detection, MRI schedules were advanced for all patients except one. All of these patients underwent wide excision and had the recurrence pathologically confirmed (Fig. 3). Subgroup analysis revealed no significant differences in the detection rate of short-term USG among subgroups based on initial tumor grade, size, depth, radiotherapy, or chemotherapy (Supplemental Table e-1).

**Table 4:** Detection rates of the routine protocol and short-term USG for diagnosing local tumor recurrence and metastatic lymph nodes

Local recurrence detection rate (%)			Metastatic lymph node detection rate (%)		
Routine protocol (n = 13)	Short-term USG (n = 7)	Total (n = 20)	Routine protocol (n = 2)	Short-term USG (n = 5)	Total (n = 7)
6.6 (3.9, 10.9)	3.5 (1.7, 7.1)	10.1 (6.6, 15.1)	1.0 (0.0, 3.6)	2.5 (1.1, 5.8)	3.5 (1.7, 7.1)

Note: Data in parenthesis indicate 95% confidence interval. USG: ultrasonography.

**Fig. 4:**
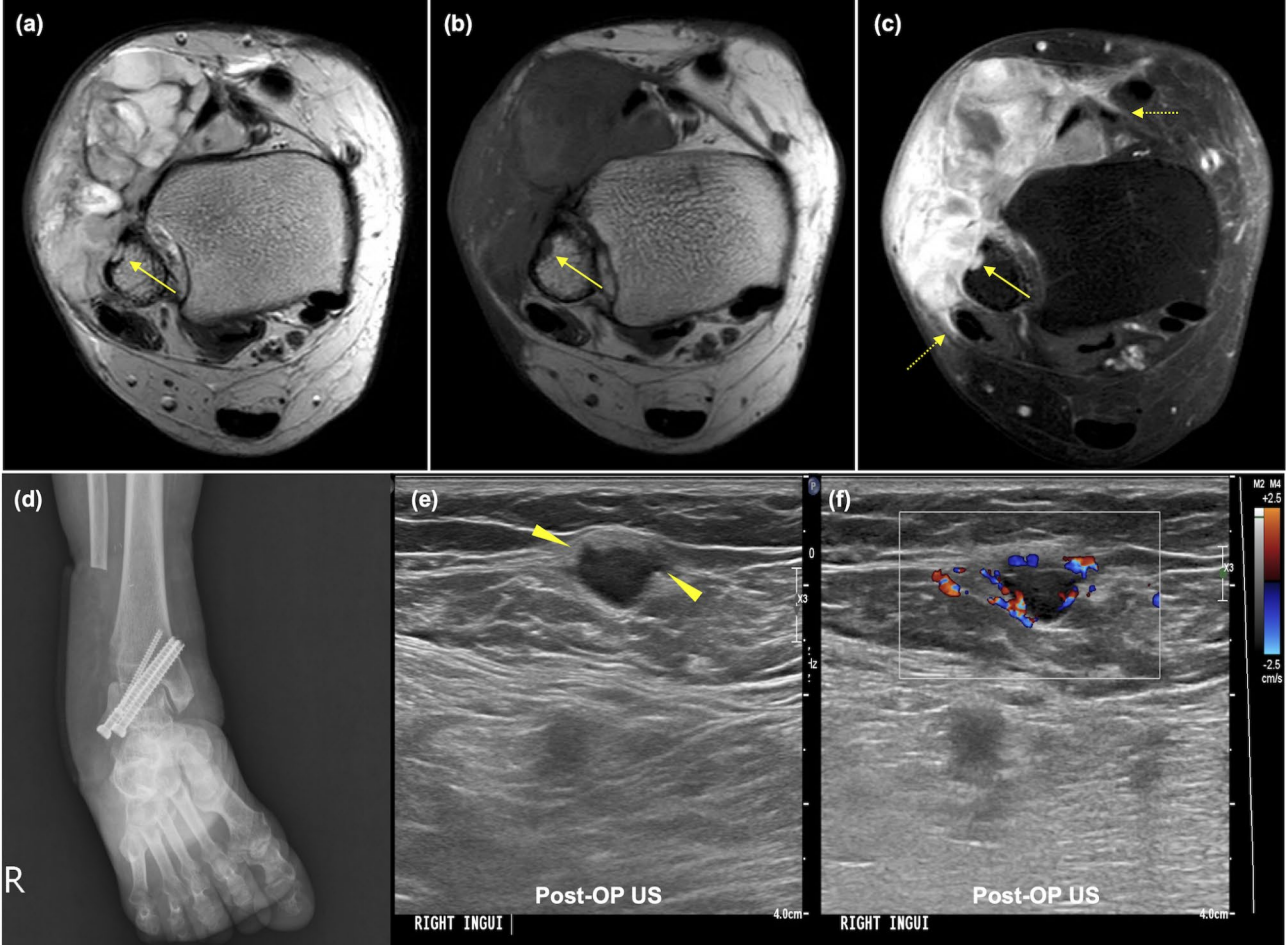
Detection of metastatic lymphadenopathy through short-term USG in a 69-year-old female with undifferentiated pleomorphic sarcoma. (**a,b,c**) Axial T2-, T1-, and enhanced T1-weighted images revealed infiltrative heterogeneous signal intensity mass with enhancement primarily located in the subcutaneous layer of the right anterolateral ankle. Focal invasion with cortical erosion at the distal fibula was noted (arrows). Additionally, the mass exhibited tail-like enhancement along the investing fascia (dashed arrows). (**d**) The patient underwent wide excision of the tumor with fibular resection and tibiotalar fusion. (**e**) On her postoperative USG, 21 months after wide resection, a small but suspicious-looking lymph node was incidentally observed in the right inguinal area. Loss of hilar echogenicity and infiltrative growth of the tumor outside the original lymph node contour were observed (arrowheads). (**f**) Color Doppler USG revealed peripheral vascularity. The lymph node was excised and confirmed as a metastatic lymph node

Short-term USG also identified five out of seven patients with newly developed metastatic lymph nodes. These metastases were located in the inguinal lymph nodes, which were not covered by MRI due to a limited field of view (Fig. 4). The yield of short-term USG for detecting metastatic lymph nodes was 2.5% (95% CI: 1.1–5.8%; 5/198).

### False referral rate of short-term USG

**Fig. 5:**
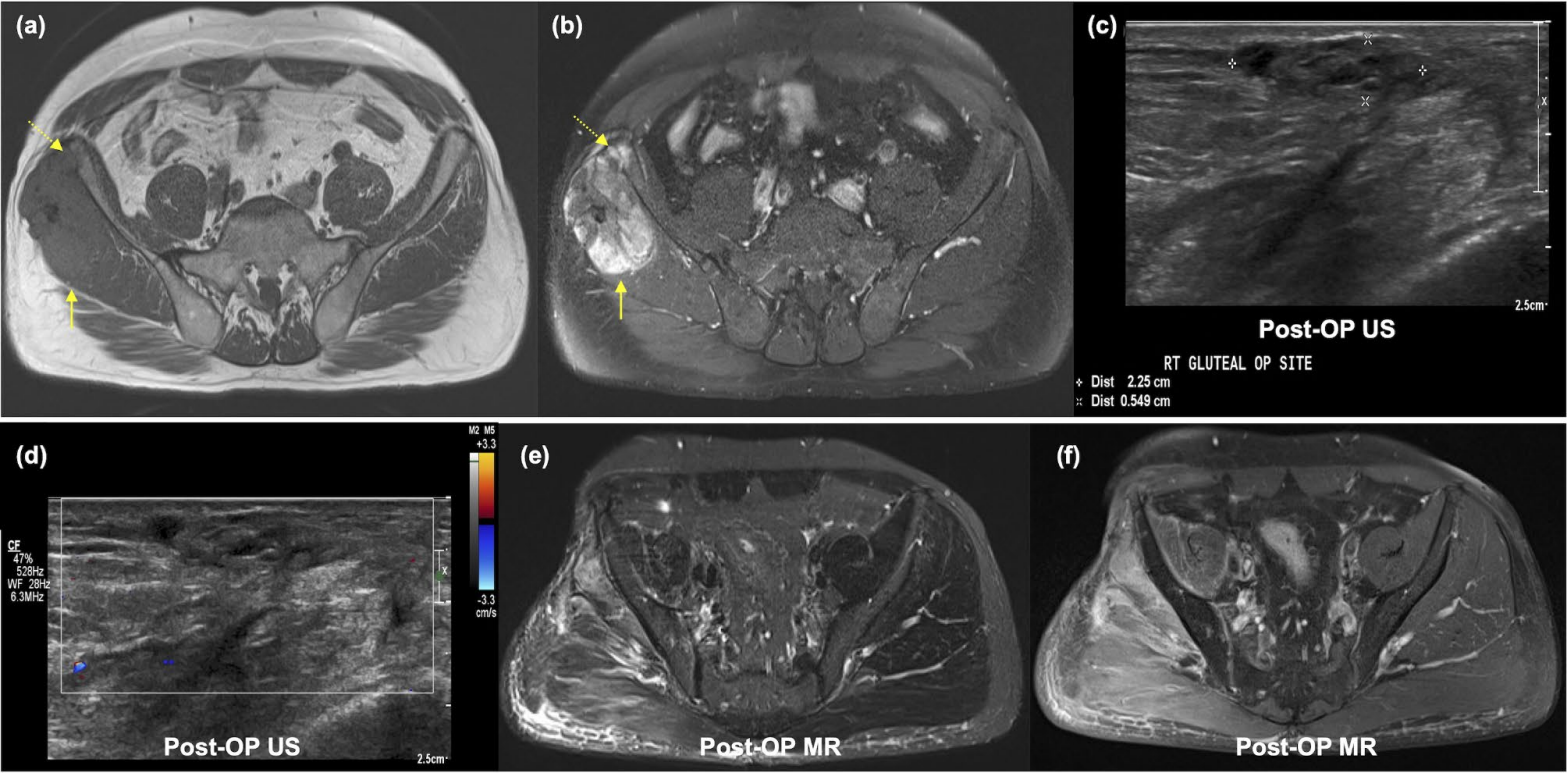
False-positive USG in a 62-year-old male with undifferentiated pleomorphic sarcoma. (**a,b**) Axial pre- and post-enhanced T1-weighted images revealed a large, heterogeneously enhancing mass (arrows) at the right gluteus medius and minimus with focal extension to the right iliac bone (dashed arrows). (**c**) On his postoperative USG, taken 9 months after wide resection, a 2.2-cm irregular ovoid-shaped lesion with internal heterogeneous echogenicity was observed in the subcutaneous layer of the right gluteus region. (**d**) No vascularity was detected based on color Doppler USG. As a result of these USG findings, the MRI examination was rescheduled. (**e,f**) Axial fat-saturated T2-weighted and enhanced T1-weighted images revealed increased signal intensity and enhancement at the right gluteus medius, gluteus minimus, and iliacus, indicative of radiation-induced myositis. However, no definite nodular-enhancing lesion corresponding to the USG findings was identified

False-positive findings were observed in 14 out of 198 patients (7.1%). Among these cases, short-term USG detected seven false-positive cases, resulting in a false referral rate of 3.5% (95% CI: 1.7–7.1%, 7/198) (Table 5).MRI schedules were adjusted for these patients; however, the MRI scans only revealed radiation-induced myositis (n = 2), traumatic neuroma (n = 1), or nonspecific postoperative changes (n = 4) (Fig. 5). None of these patients experienced true recurrence during the remaining follow-up period.

**Table 5:** False referral rates of the routine protocol and short-term USG.

False referral rate (%)		
Routine protocol (n = 7)	Short-term USG (n = 7)	Total (n = 14)
3.5 (1.7, 7.1)	3.5 (1.7, 7.1)	7.1 (5.3, 11.5)

Note: Data in parenthesis indicate 95% confidence interval. USG: ultrasonography.

## Discussion

In this study, we demonstrated the additional benefit of incorporating short-term USG for early detection of local recurrence of STS and identification of missed metastatic lymph nodes when using MRI alone.

Our findings contradict previous studies suggesting that intensive imaging follow-up for STS is unnecessary [4, 8, 9]. Such studies found no significant difference in the overall detection rate of local recurrence between intensive and routine follow-up protocols [8, 10]. However, we suggest that comparing the overall detection rate alone does not provide sufficient clinical significance. For example, if a group undergoes both intensive 3-month-interval and routine 6-month-interval protocols, any local recurrence detected through the intensive protocol would also be detected later through the routine protocol, resulting in no difference in the overall detection rate between the protocols. Therefore, instead of focusing on overall detection rate, we aimed to determine how early intensive follow-up can detect local recurrence compared with the routine protocol in a single cohort of patients. We found that seven patients experienced local recurrences that were detected through short-term USG prior to their routine MRI visits, resulting in an additional yield of 3.5% for early detection (7/198 patients). Following detection through short-term USG, the MRI schedules for all patients, except one, were advanced. The remaining patient underwent routine MRI as scheduled because the size of the local recurrence on USG (0.8 cm) was too small to make a confident decision. Surgical resection was performed in all patients, confirming the presence of local recurrence. Therefore, the incorporation of short-term USG aided in the early detection of local tumor recurrence.

A previous prospective comparative study found that USG and MRI shared a similar local recurrence detection performance [16]. Based on these findings, we suggest that it is appropriate to use either MRI or USG as intensive follow-up imaging methods in STS surveillance. However, USG offers several advantages, including lower cost, ease of use, and shorter scan time. Consequently, we incorporated short-term USG into our routine MRI follow-up protocol. Another advantage of USG over MRI is its ability to cover various remote regions, making it more suitable for detecting metastases. In our institution, USG exams routinely include scanning of popliteal, inguinal, epitrochlear, or axillary lymph nodes, depending on the primary tumor locations. Our findings revealed that short-term USG detected five additional patients with new metastatic lymph nodes that were outside the field of view of MRI. When combining the cases of local recurrence and metastatic lymph nodes, 12 out of 198 patients (6.0%) benefited from the inclusion of short-term USG in our surveillance protocol.

Regarding the 20 patients with local recurrence, the median size of the recurrent tumors was 1.7 cm (range: 0.8–6.0 cm). Twelve of the recurrences were superficially located, whereas eight were deeply seated. Most local recurrences exhibited well-defined hypoechoic masses, with only three cases showing slightly ill-defined margins. None of the local recurrences displayed fascial thickening as a sole feature of relapse. Notably, the use of physical examination resulted in the detection of local recurrence in only 6 (30%) of these 20 patients, which contradicts previous studies highlighting the importance of physical examination for detecting local recurrence [4, 8, 9]. However, the previous studies did not provide explicit details on the regularity of imaging follow-up, frequency of imaging studies, or the specific imaging modality employed. If regular local imaging was not performed, it is possible that recurrent tumors could remain undetected until they reach a size that is detectable through palpation. Given that physical examination failed to detect local recurrence in the remaining 14 patients who were found to have recurrent tumors using either USG or MRI, we can assume that regular imaging surveillance increases the likelihood of early tumor progression identification compared with physical examination alone. Indeed, a recent study demonstrated that the size of locally recurrent tumors detected through regular MRI surveillance was significantly smaller than that of tumors detected through non-MRI surveillance, including physical examination (2.3 ± 1.3 cm vs. 4.0 ± 3.4 cm; *P* = 0.001) [17]. Similarly, the median size of recurrent tumors in our study (1.7 cm) was smaller than that in previous studies [17, 18]. Notably, the median size of recurrent tumors detected through short-term USG was even smaller (1.5 cm, range: 0.8-3.0 cm).

Subgroup analysis did not identify any clinical or tumor characteristics that influenced the detection rate of short-term USG. This may be due to the limited number of local recurrences in our study, which was insufficient to derive statistically significant results [19]. We speculate that the detection rate of USG might vary depending on the location of the tumor, as USG is more effective at detecting superficially located tumors, as suggested by the NCCN guidelines and previous studies [7, 20, 21]. In our study, the detection rate of short-term USG was higher for tumors located in the dermis or subcutaneous layer compared with those located in the intramuscular or intermuscular layer, although statistical significance was not observed (4.2% vs. 2.9%; *P* = 0.71). Additionally, the detection rate of short-term USG was higher for tumors smaller than 5 cm compared with larger tumors (≥ 5 cm), although no statistical significance was observed. Further studies with larger cohorts are warranted to validate our findings.

Short-term USG yielded 7 false-positive cases out of the total 14 false-positive cases. All of these cases showed hypoechoic masses, with five out of seven demonstrating well-defined margins that were indistinguishable from true recurrences based on USG features. MRI schedules were advanced in these seven patients and MRI examinations confirmed no tumor recurrence: four exhibited postoperative changes, two exhibited radiation myositis, and one exhibited a traumatic neuroma. None of these patients underwent unnecessary invasive procedures.

Our study has several limitations. First, only 198 patients were included, given the rarity of STS and the exclusion of low-grade STS, of which 20 experienced local recurrences. Low-grade STS was excluded because it shows infrequent recurrences and generally requires a longer surveillance period (10 years) for local recurrence detection [22, 23]. Nevertheless, as our institution is one of the largest tertiary referral hospitals for STS in the country, we extended the recruitment period to 10 years to include as many eligible patients as possible. Second, due to retrospective nature of the study, a substantial number of patients did not complete our intensive surveillance protocol (n = 75). We compared the characteristics of patients who completed the follow-up protocol and those who did not (Supplemental Table e-2), with the former being more likely to have tumors in superficial locations (48.0% vs. 28.8%; *P* = 0.004) and smaller tumor sizes (6.6 ± 4.2 cm vs. 8.6 ± 5.2 cm; *P* = 0.004). This difference may be attributed to larger tumors at initial presentation displaying more aggressive behavior, such as distant metastasis, leading to more frequent follow-up loss before protocol completion [24]. Additionally, patients who experienced superficial palpable tumors may have been more motivated to undergo follow-up examinations because these patients were more alert to any palpable lesion in fear of local recurrence. Another reason for incomplete protocol adherence was the preference of physicians or patients to attend follow-up visits in other hospitals closer to their residence. No significant differences were observed in other characteristics between the two groups. Third, only static USG images were available for the analysis. Due to operator-dependency of USG, some small recurrences could have been undetected at the first USG scan or there could have been an increase or decrease in false positive results. This might have degraded our study results. However, double checking by the staff members was always performed. Therefore, missed cases or false positive results could be minimized. In addition, to overcome the limitation of static USG images, we performed consensus reading instead of inter-reader agreement analysis. Fourth, we did not evaluate whether the early detection of local recurrence improved overall survival, which is a crucial clinical consideration. A previous study reported that early detection of local recurrence improved disease-specific survival [11]. However, a well-controlled prospective study is required to validate this finding. Finally, we did not perform a cost-effectiveness analysis of short-term USG. Nevertheless, out of the 20 patients with local recurrence, 7 patients (35%) benefited from short-term USG. Although short-term USG increased the number of false-positive cases, none of the patients underwent invasive procedures.

## Conclusion

Short-term USG for postoperative surveillance of high-grade STS can enhance early detection of local tumor recurrence and metastatic lymphadenopathy. Early detection of local tumor recurrence could lead to a prompt surgical resection and aid in local disease control.

### Electronic supplementary material

Below is the link to the electronic supplementary material.

Supplementary Material 1

Supplementary Material 2

Supplementary Material 3

## Data Availability

The datasets used and/or analyzed during the current study are available from the corresponding author on reasonable request.

## Abbreviations

USG: Ultrasonography
MRI: Magnetic resonance imaging
STS: Soft tissue sarcoma
