# Supplementary material for: Enhancing local recurrence detection in patients with high-grade soft tissue sarcoma: value of short-term Ultrasonography added to post-operative MRI surveillance
Source: Cancer Imaging. 2024 Jan 19;24:12. doi: 10.1186/s40644-023-00645-9 (PMC10797984; doi:10.1186/s40644-023-00645-9)
Supplement: Supplementary file 3 — Supplementary Material 3 [file 40644_2023_645_MOESM3_ESM.docx]

**Supplemental Table e-1. Detection rate of short-term USG according to patient subgroups.**

| Subgroups | Detection rate of short-term USG (%) | *P*-value |
| --- | --- | --- |
| Grade |  | 0.71 |
| Grade 2 (n = 122) | 4.1 (1.7, 9.2) |  |
| Grade 3 (n = 76) | 2.6 (0.7, 9.0) |  |
| Size |  | 0.13 |
| $<$5 cm (n = 94) | 5.3 (2.3, 11.9) |  |
| 5 –10 cm (n = 62) | 0 (0.0, 5.8) |  |
| 10 –15 cm (n = 30) | 0.03 (0.0, 16.7) |  |
| $\geq$15 cm (n = 12) | 0.08 (1.4, 35.4) |  |
| Depth |  | 0.71 |
| Intra- or intermuscular (n = 103) | 2.9 (1.0, 8.2) |  |
| Dermis or subcutaneous (n = 95) | 4.2 (1.6, 10.3) |  |
| Radiation therapy |  | > 0.99 |
| Negative (n = 65) | 3.1 (0.8, 10.5) |  |
| Positive (n = 133) | 3.8 (1.6, 8.5) |  |
| Chemotherapy |  | 0.68 |
| Negative (n = 134) | 3.0 (1.2, 7.4) |  |
| Positive (n = 64) | 4.7 (1.6, 12.9) |  |
| Note: Data in parenthesis indicate 95% confidence interval. USG: ultrasonography. | | |

**Supplemental Table e-2. Characteristics of patients who did and did not undergo the complete follow-up protocol.**

| Characteristics | Complete protocol (n = 198) | Incomplete protocol (n = 73) | *P*-value |
| --- | --- | --- | --- |
| Sex |  |  | 0.74 |
| Female | 104 (52.5) | 36 (49.3) |  |
| Male | 94 (47.5) | 37 (50.7) |  |
| Age (years) | 52.1 ± 15.8 | 56.2 ± 20.6 | 0.12 |
| Type of sarcoma |  |  | 0.50 |
| UPS | 63 (31.8) | 24 (32.9) |  |
| Myxoid LPS | 29 (14.6) | 6 (8.2) |  |
| MFS | 27 (13.6) | 9 (12.3) |  |
| Etc. | 79 (39.9) | 34 (46.6) |  |
| Location |  |  | 0.07 |
| Lower extremity | 131 (66.2) | 58 (79.5) |  |
| Upper extremity | 35 (17.7) | 10 (13.7) |  |
| Trunk | 32 (16.2) | 5 (6.8) |  |
| Depth |  |  |  |
| Intra or intermuscular | 103 (52.0) | 52 (71.2) | 0.004 |
| Dermis or subcutaneous | 95 (48.0) | 21 (28.8) |  |
| Size (cm) | 6.6 ± 4.2 | 8.6 ± 5.2 | 0.004 |
| Radiotherapy |  |  | 0.48 |
| No treatment | 65 (32.8) | 28 (38.4) |  |
| Treatment | 133 (67.2) | 45 (61.6) |  |
| Chemotherapy |  |  | 0.47 |
| No treatment | 134 (67.7) | 46 (63.0) |  |
| Treatment | 64 (32.3) | 27 (37.0) |  |
| Note: Data in parenthesis indicate percentages. UPS: undifferentiated pleomorphic sarcoma; LPS: liposarcoma; MFS: myxofibrosarcoma. | | | |
